# Evidence for transcriptional interference in a dual-luciferase reporter system

**DOI:** 10.1038/srep17675

**Published:** 2015-12-01

**Authors:** Guo-Qing Wu, Xiao Wang, Hong-Ying Zhou, Ke-Qun Chai, Qian Xue, Ai-Hong Zheng, Xiu-Ming Zhu, Jian-Yong Xiao, Xu-Hua Ying, Fu-Wei Wang, Tao Rui, Li-Yun Xu, Yong-Kui Zhang, Yi-Ji Liao, Dan Xie, Li-Qin Lu, Dong-Sheng Huang

**Affiliations:** 1 Department of Oncology & Cancer Biotherapy Center, Zhejiang Provincial People’s Hospital, 158 Shangtang Road, Hangzhou, Zhejiang 310014, China; 2 Zhejiang Academy of Traditional Chinese Medicine, Tongde Hospital of Zhejiang Province, 234 Gucui Road, Hangzhou, Zhejiang 310012, China; 3 Department of Biochemistry, Guangzhou University of Chinese Medicine, 232 Waihuang Road East, Guangzhou, Guangdong 510006, China; 4 Cell and Molecular Biology Laboratory, Zhoushan Hospital, Zhoushan, Zhejiang 316000, China; 5 Department of Cardio-Thoracic Surgery, Zhoushan Hospital, Zhoushan, Zhejiang 316000, China; 6 State Key Laboratory of Oncology in South China, Cancer Center, Sun Yat-Sen University, 651 Dongfeng Road East, Guangzhou, Guangdong 510060, China

## Abstract

The dual-luciferase reporter assay is widely used for microRNA target identification and the functional validation of predicted targets. To determine whether curcumin regulates expression of the histone methyltransferase enhancer of zeste homolog 2 (EZH2) by targeting its 3′untranslated region (3′UTR), two luciferase reporter systems containing exactly the same sequence of the EZH2 3′UTR were used to perform dual-luciferase reporter assays. Surprisingly, there were certain discrepancies between the luciferase activities derived from these two reporter constructs. We normalized luciferase activity to an internal control to determine the amount of the reporter construct successfully transfected into cells, induced a transcriptional block with flavopiridol, quantified renilla luciferase mRNA levels, and compared the absolute luciferase activity among the different groups. The results suggested that curcumin promoted the transcription of the luciferase genes located downstream of the simian vacuolating virus 40 (SV40) early enhancer/promoter, but not those located downstream of the human cytomegalovirus (CMV) immediate-early or the herpes simplex virus thymidine kinase (HSV-TK) promoters. These results explain the discrepancies between the two luciferase reporter systems. The current study underscores the importance of taking caution when interpreting the results of dual-luciferase reporter assays and provides strategies to overcome the potential pitfall accompanying dual-luciferase reporter systems.

The luciferase reporter assay is a standard method used to study mRNA processing and the expression of microRNA (miRNA) targets. Dual-luciferase reporter systems utilize firefly and renilla luciferase, which are introduced into cells either by transfecting cells with a dual-luciferase reporter construct or by co-transfecting cells with a luciferase reporter construct and an internal control vector and are well known to improve experimental accuracy. Curcumin is well known for its anti-cancer effects. In a previous study, we found that curcumin inhibits lung cancer cell proliferation by down-regulating the expression of enhancer of zeste homolog 2 (EZH2) and up-regulating the expression levels of miR-101 and miR-let 7c (Wu, G-Q *et al*. submitted for publication). These miRNAs are believed to be tumor-suppressor miRNAs that down-regulate EZH2 expression by binding to the EZH2 3′ untranslated region (3′UTR) in a variety of cancer cells^1^,^2^,^3^,^4^,^5^,^6^,^7^. To determine whether curcumin regulates the expression of EZH2 by inducing miR-101 and miR-let 7c expression, two luciferase reporter systems containing exactly the same sequence of the EZH2 3′UTR were used to perform dual-luciferase reporter assays. However, contradictory results were obtained with these two luciferase reporter systems. In the present study, we explored the underlying causes of the conflicting results, and identified alternative measures to compensate for the anomalous results we had observed using the two different dual-luciferase reporter systems.

## Results

### Contradictory results derived from two luciferase reporter systems

**Figure 1:**
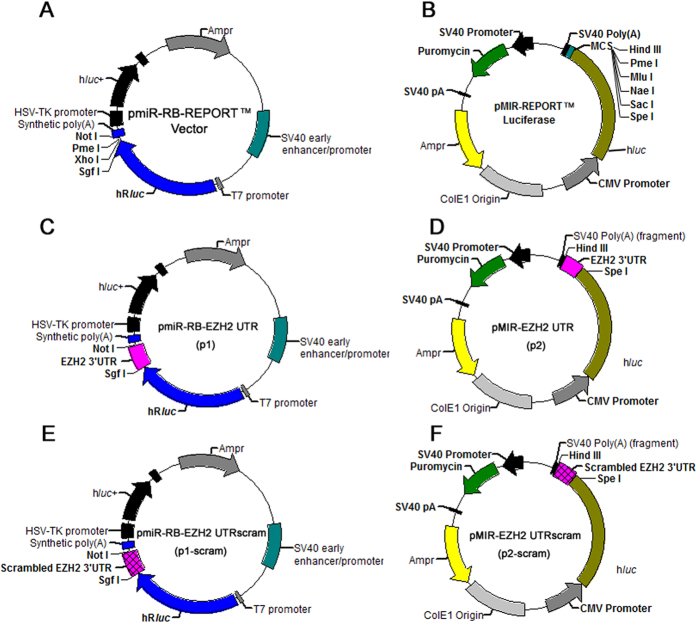
Luciferase reporter vectors. Abbreviations: Ampr, ampicillin resistance gene; h*luc*+ or h*luc*, *firefly luciferase* gene; hR*luc*, *renilla luciferase* gene; SV40 early enhancer/promoter, simian vacuolating virus 40 early enhancer/promoter; CMV Promoter, human cytomegalovirus immediate-early promoter.

**Figure 2:**
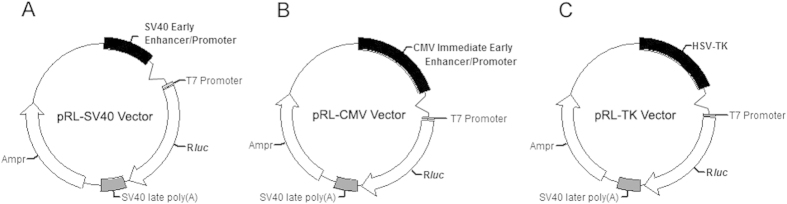
Control vectors. Abbreviations: Ampr, ampicillin resistance gene; R*luc*, *renilla luciferase* gene; SV40 early enhancer/promoter, simian vacuolating virus 40 early enhancer/promoter; CMV Promoter, human cytomegalovirus immediate-early promoter; HSV-TK, herpes simplex virus thymidine kinase promoter.

**Figure 3:**
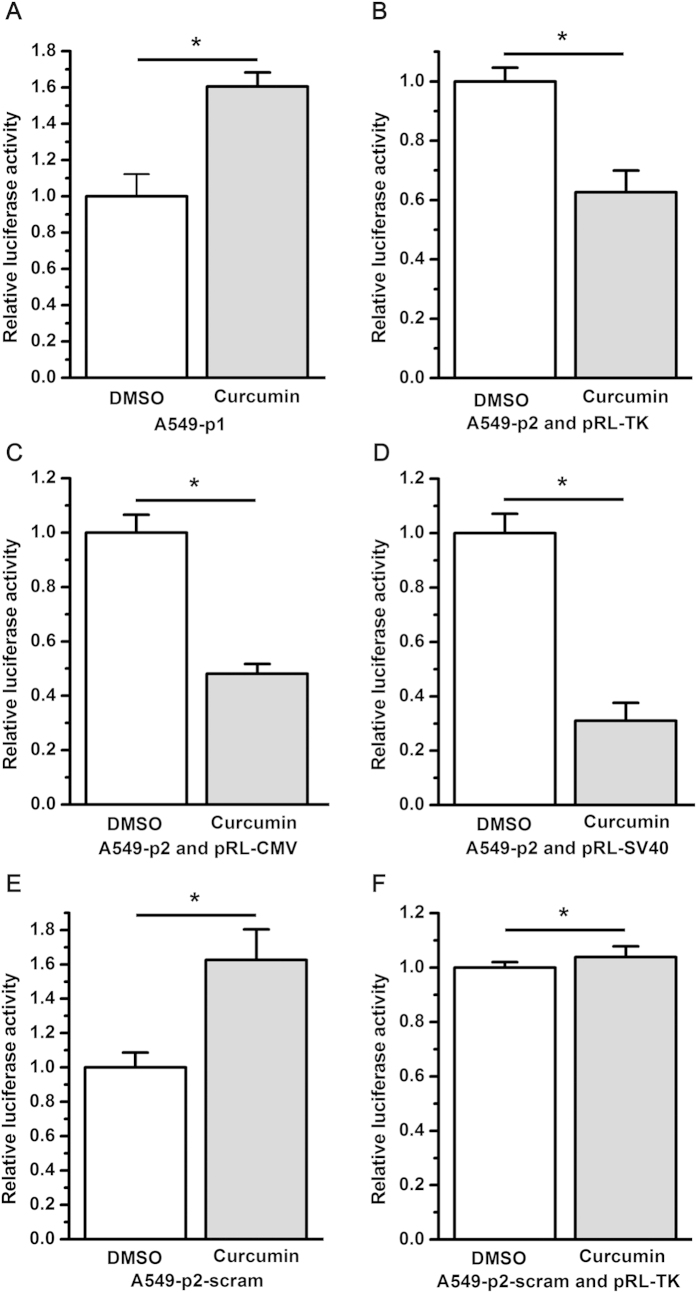
Different luciferase reporter systems provided conflicting data regarding the effect of curcumin on the EZH2 3′UTR. (**A**) Curcumin treatment led to increased luciferase activity relative to DMSO treatment as determined by the dual-luciferase reporter assay carried out with p1 (*P < 0.05). (**B**–**D**) Curcumin treatment led to a significant decrease in luciferase activity relative to DMSO treatment as determined by the luciferase reporter assay performed with p2 and the control vector pRL-TK, pRL-CMV, or pRL-SV40 (*P < 0.05). (**E**) Curcumin treatment led to increased luciferase activity relative to DMSO treatment as determined by the luciferase reporter assay carried out with p1-scram (*P < 0.05). (**F**) Compared to the control treatment with DMSO, treatment with curcumin did not affect luciferase activity when the luciferase reporter assay was performed using p2-scram and the control vector pRL-TK (N/S, not statistically significant; *P* > 0.05). For each EZH2 3′UTR or scrambled EZH2 3′UTR, luciferase activity (hRluc:hluc or hluc:hRluc) was normalized to 1 relative to the control treatment with DMSO. The data in all of the bar graphs were plotted as the mean ± SEM.

To determine whether curcumin regulates EZH2 expression by targeting the 3′UTR of EZH2, luciferase reporter assays were performed with EZH2 3′UTR reporters. Because it was previously reported that the activity of the internal control plasmid can be affected by the presence of co-transfected reporter plasmids used to normalize the transfection efficiency^8^, we employed two dual-luciferase reporter systems containing exactly the same sequence of the EZH2 3′UTR. To construct the EZH2 3′UTR reporter systems, 263 bp of the EZH2 3′UTR was inserted into the dual-luciferase reporter vector pmiR-RB-REPORT^TM^ between the Sgf I and Not I sites (Fig. 1A), or into the luciferase reporter vector pMIR-REPORT^TM^ Luciferase between the Spe I and Hind III sites (Fig. 1B) to generate pmiR-RB-EZH2 UTR (p1) (Fig. 1C) and pMIR-EZH2 UTR (p2) (Fig. 1D), respectively. A scrambled sequence of the EZH2 3′UTR was inserted into the dual-luciferase reporter vector pmiR-RB-REPORT^TM^ or into the luciferase reporter vector pMIR-REPORT^TM^ Luciferase to generate the negative control constructs pmiR-RB-EZH2 UTRscram (p1-scram) (Fig. 1E) and pMIR-EZH2 UTRscram (p2-scram) (Fig. 1F), respectively. A549 cells were then transfected with the dual-luciferase reporter construct p1, or co-transfected with the luciferase reporter construct p2 and the control vector pRL-TK, pRL-SV40, or pRL-CMV (Fig. 2A–C). Unexpectedly, compared with dimethylsulfoxide (DMSO), curcumin caused a significant increase in EZH2 3′UTR-renilla luciferase activity in A549 cells transfected with the dual-luciferase reporter construct p1 containing the EZH2 3′UTR (Fig. 3A). In contrast, in A549 cells co-transfected with the luciferase reporter construct p2 and the control vector pRL-TK, pRL-CMV, or pRL-SV40, there was a significant reduction in EZH2 3′UTR-firefly luciferase activity in cells treated with curcumin relative to those treated with DMSO (Fig. 3B–D), which is in agreement with the finding that curcumin inhibited EZH2 mRNA and protein expression. Luciferase reporter assay results with NCI-H2170 cells are consistent with that of A549 cells (data not shown).

To explore the causes of the conflicting results obtained with the two luciferase reporter systems, A549 cells were transfected with the negative control dual-luciferase reporter construct p1-scram, or co-transfected with the negative control luciferase reporter construct p2-scram and the internal control vector pRL-TK. Unexpectedly, curcumin increased scrambled EZH2 3′UTR-renilla luciferase activity in A549 cells transfected with p1-scram (Fig. 3E). However, scrambling the EZH2 3′UTR sequence in the luciferase reporter construct p2 completely abrogated the inhibitory effects of curcumin on firefly luciferase activity (Fig. 3F).

### The regulation of gene transcription by curcumin is driven by different types of promoters

**Figure 4:**
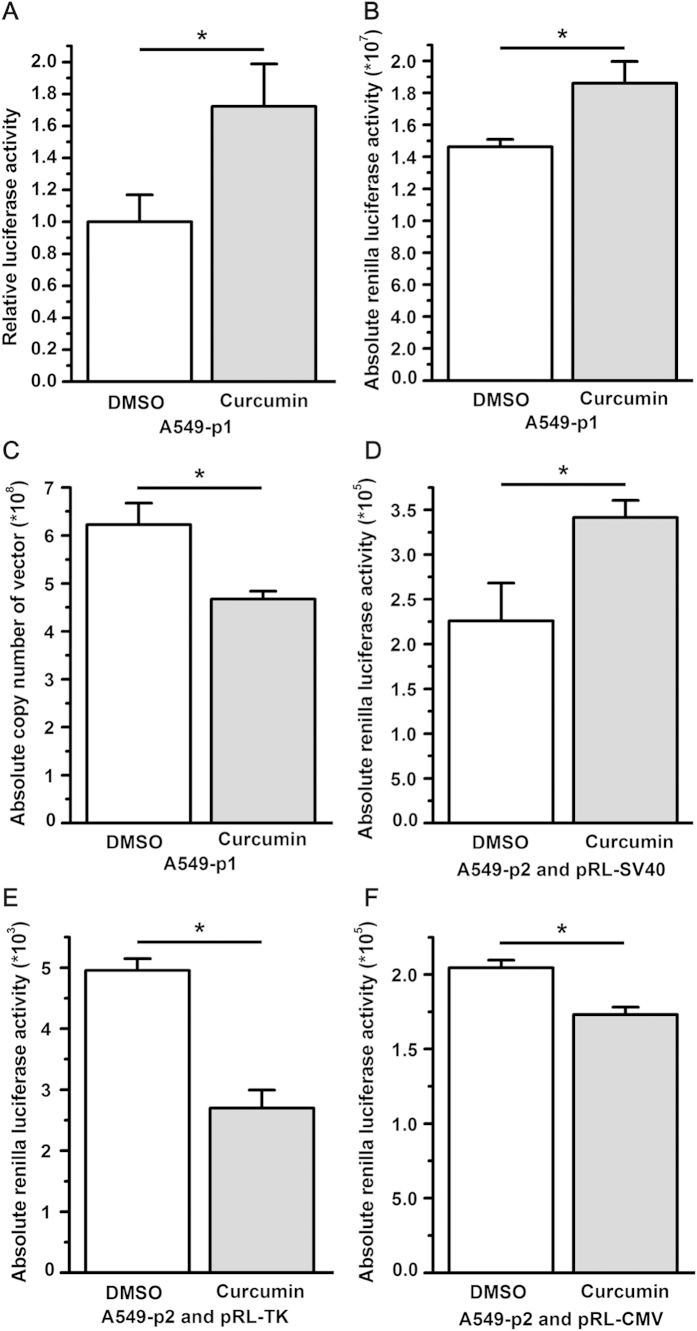
Curcumin promoted the transcription of the *luciferase* gene located downstream of the SV40 early enhancer/promoter. (**A**–**C**) Curcumin treatment increased renilla luciferase activity relative to DMSO treatment when the absolute copy number of the dual-luciferase reporter vector p1 successfully transfected into A549 cells was used as an internal control (**A**, *P < 0.05). Luciferase activity (hRluc:absolute copy number of vector) was normalized to 1 relative to the control treatment with DMSO. The absolute renilla luciferase activity derived from A549 cells treated with curcumin was higher than that derived from A549 cells treated with DMSO (**B**, *P < 0.05). The absolute copy number of the dual-luciferase reporter vector p1 successfully transfected into A549 cells treated with curcumin was lower than that of the A549 cells treated with DMSO (**C**, *P < 0.05). (**D–F**) When pRL-SV40 was used as the internal control, the absolute renilla luciferase activity derived from A549 cells treated with curcumin was higher than that derived from A549 cells treated with DMSO (**D**, *P < 0.05). When pRL-TK or pRL-CMV was used as the internal control, the absolute renilla luciferase activity derived from A549 cells treated with curcumin was lower than that derived from A549 cells treated with DMSO (**E** and F, *P < 0.05). The data in all of the bar graphs were plotted as the mean ± SEM.

The two different luciferase reporter systems generated contradictory data regarding the effects of curcumin on luciferase reporter expression. To reconcile this discrepancy, we focused on the dual-luciferase reporter vector pmiR-RB-REPORT^TM^. Based on the analysis of the features of this vector, several possibilities were proposed to explain the inconsistent results. First, we speculated that the activity of the internal control firefly luciferase in p1 was suppressed by curcumin, which makes it unable to serve as an internal control. To test this hypothesis, quantitative real-time PCR (qPCR) was employed to determine the absolute copy number of the dual-luciferase reporter construct p1 that was successfully transfected into the A549 cells. The specific activity of EZH2 3′UTR-renilla luciferase in cells treated with curcumin was compared to that in cells treated with DMSO by normalizing to the absolute copy number of the internal control plasmid. In agreement with the results generated by using firefly luciferase activity as the internal control, the EZH2 3′UTR-renilla luciferase activity derived from the dual-luciferase reporter construct p1 was higher in cells treated with curcumin compared to those treated with DMSO (Fig. 4A). This indicates that curcumin likely does not affect the expression of the *firefly luciferase* gene driven by the herpes simplex virus thymidine kinase (HSV-TK) promoter in p1.

Second, curcumin might facilitate the transcription of the *renilla luciferase* located downstream of the simian vacuolating virus 40 (SV40) early enhancer/promoter in p1, resulting in increased renilla luciferase activity in A549 cells treated with curcumin relative to those treated with DMSO. To test this possibility, the viable cell number, the absolute copy number of p1 that was successfully transfected, and the absolute renilla luciferase activity were compared in A549 cells treated with either curcumin or DMSO 48 hours post-transfection. In agreement with the hypothesis, the absolute EZH2 3′UTR-renilla luciferase activity was higher in A549 cells treated with curcumin compared to those treated with DMSO (Fig. 4B), despite the fact that the number of viable A549 cells and the absolute copy number of p1 in A549 cells treated with curcumin were much lower than in cells treated with DMSO (Fig. 4C). This result strongly points to the stimulatory effect of curcumin on the transcription of EZH2 3′UTR-*renilla luciferase* located downstream of the SV40 early enhancer/promoter.

Because the expression of *renilla luciferase* in the control vector pRL-SV40 is also driven by the SV40 early enhancer/promoter as shown in Fig. 2, and to validate the effect of curcumin on the SV40 early enhancer/promoter, we also determined renilla luciferase activity in curcumin- or DMSO-treated A549 cells co-transfected with p2 and pRL-SV40. In agreement with the data from p1, the absolute renilla luciferase activity in A549 cells treated with curcumin was significantly higher than that in cells treated with DMSO (Fig. 4D) despite a lower number of viable cells and a lower copy number of the control vector pRL-SV40 in the curcumin group. In contrast to the data from pRL-SV40, when A549 cells were co-transfected with p2 and the control vector pRL-TK or pRL-CMV, the absolute renilla luciferase activity following curcumin treatment was significantly lower than that following treatment with DMSO (Fig. 4E,F). As shown in Fig. 2, the main difference between these control vectors is the type of promoter and/or enhancer that drives the transcription of *renilla luciferase*. Thus, the results indicate that the SV40 early enhancer/promoter contributes to higher renilla luciferase activity in curcumin-treated A549 cells co-transfected with p2 and pRL-SV40 relative to those treated with DMSO.

### Overexpression of miR-let 7c and miR-101 inhibits EZH2 3′UTR-luciferase activity

**Figure 5:**
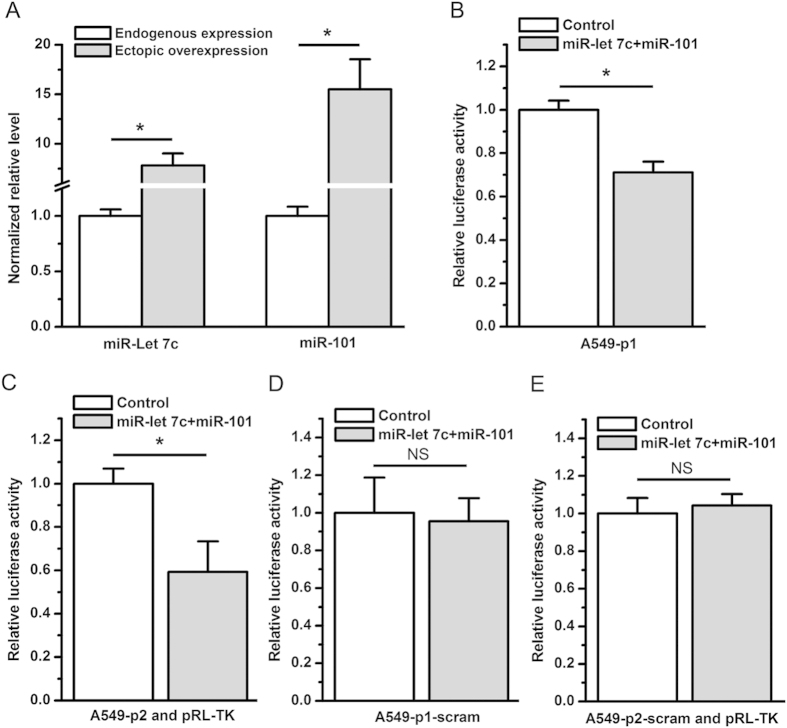
miR-let 7c and miR-101 inhibited EZH2 3′UTR-luciferase activity. (**A**) The expression of miR-let 7c and miR-101 in A549 cells was significantly elevated after the co-transduction of LV-miR-let 7c and LV-miR-101 (*P < 0.05). (**B**) Compared with LV-Control, LV-miR-let 7c and LV-miR-101 inhibited EZH2 3′UTR-renilla luciferase activity from p1 (*P < 0.05). (**C**) Compared with LV-Control, LV-miR-let 7c and LV-miR-101 inhibited EZH2 3′UTR-firefly luciferase activity from p2 (*P < 0.05). (**D**) Compared with LV-Control, LV-miR-let 7c and LV-miR-101 did not affect the luciferase activity when the luciferase reporter assay was carried out with p1-scram (NS, not statistically significant). (**E**) Compared with LV-Control, LV-miR-let 7c and LV-miR-101 did not alter luciferase activity when the luciferase reporter assay was carried out with p2-scram and the control vector pRL-TK (NS, not statistically significant). The luciferase activity (hRluc:hluc or hluc:hRluc) in A549 cells co-transduced with LV-miR-let 7c and LV-miR-101 was normalized to 1 relative to the luciferase activity in cells transduced with LV-Control. The data in all of the bar graphs were plotted as the mean ± SEM.

To further evaluate whether miR-let 7c and miR-101 regulate EZH2 3′UTR-luciferase activity in A549 cells, we co-transduced A549 cells with precursor miRNAs (LV-miR-let 7c and LV-miR-101) or control precursor miRNA (LV-Control). As determined by qPCR, the co-transduction of LV-miR-let 7c and LV-miR-101 induced a 7- and 15-fold overexpression of miR-let 7c and miR-101, respectively (Fig. 5A). In agreement with previous reports^1^,^2^,^3^,^4^,^5^,^6^,^7^, the overexpression of miR-let 7c and miR-101 significantly inhibited EZH2 3′UTR-luciferase activity in A549 cells regardless of whether the assay was carried out with p1 or p2 (Fig. 5B,C). Scrambling the EZH2 3′UTR sequence in the luciferase reporter construct p1 or p2 completely abrogated the inhibitory effect of miR-let 7c and miR-101 on luciferase activity (Fig. 5D,E).

### Curcumin does not affect the decay of renilla luciferase mRNA transcribed from *renilla luciferase* located downstream of the SV40 early enhancer/promoter in pRL-SV40

**Figure 6:**
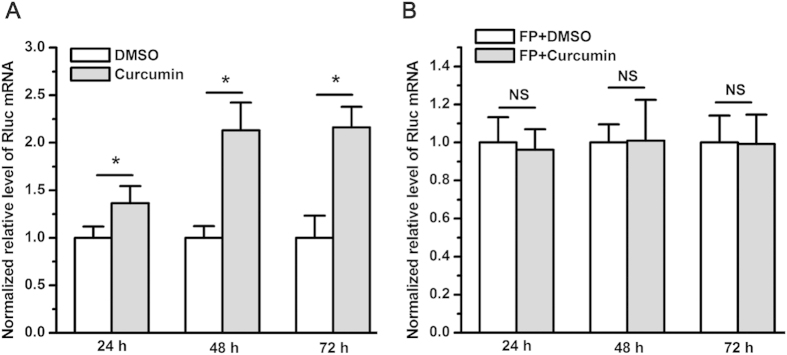
Curcumin did not affect the decay of renilla luciferase mRNA transcribed from pRL-SV40. (**A**) Curcumin significantly increased renilla luciferase mRNA levels compared with DMSO (*P < 0.05). (**B**) Two hundred nM of FP completely abrogated the curcumin-mediated increase in renilla luciferase mRNA levels (NS, not statistically significant). The renilla luciferase mRNA level was normalized to that of ACTB and subsequently normalized to 1 relative to the control treatment with DMSO. The data in all of the bar graphs were plotted as the mean ± SEM.

To examine the effect of curcumin on renilla luciferase mRNA levels when the *renilla luciferase* gene lies downstream of the SV40 early enhancer/promoter, qPCR was carried out on pRL-SV40-transfected A549 cells treated with curcumin or DMSO. In agreement with the data from the luciferase reporter assay, qPCR revealed that, compared with DMSO, curcumin significantly increased renilla luciferase mRNA levels in A549 cells transfected with pRL-SV40 (Fig. 6A). To further determine the contribution of RNA decay to renilla luciferase transcript levels in A549 cells treated with curcumin, flavopiridol (FP) was used to block gene transcription, and qPCR was employed to determine renilla luciferase mRNA levels. The results demonstrated that 200 nM of FP completely abrogated the enhancement of renilla luciferase mRNA levels by curcumin (Fig. 6B), despite the fact that it significantly inhibited cell proliferation when combined with curcumin (Supplementary Fig. S1A,B). In addition, FP caused a time-dependent decrease in renilla luciferase mRNA levels (Supplementary Fig. S1C), which indicates that FP blocks the transcription of *renilla luciferase*. This is in line with previous reports showing that FP is a potent inhibitor of gene transcription^9^,^10^.

## Discussion

This is the first report demonstrating opposing transcriptional effects on the expression of the reference and experimental reporter luciferases. We hypothesized that either the activity of the promoter driving the transcription of *renilla luciferase* or the expression of the internal control firefly luciferase was altered by curcumin. Using the absolute copy number of the dual-luciferase reporter construct p1 transfected into A549 cells as an internal control, we precluded the possibility that curcumin affected the expression of firefly luciferase from the pmiR-RB-REPORT^TM^ Vector.

Although the number of viable A549 cells and the absolute copy number of p1 in A549 cells treated with curcumin were much lower than those in cells treated with DMSO, the absolute EZH2 3′UTR-renilla luciferase activity was higher in A549 cells treated with curcumin compared with those treated with DMSO, indicating that curcumin regulates the SV40 promoter activity of p1. The renilla luciferase activity derived from pRL-SV40 was much higher in cells treated with curcumin than in cells treated with DMSO, but this was not the case when cells were transfected with the control vector pRL-TK or pRL-CMV, further corroborating the finding that curcumin regulates SV40 promoter activity, but not TK or CMV promoter activity.

In agreement with the results of the absolute renilla luciferase activity, we demonstrated that when pRL-TK or pRL-CMV was used as the internal control, curcumin inhibited EZH2 3′UTR-firefly luciferase activity approximately 40–50%. The inhibition rate was approximately 70% when pRL-SV40 was used as the internal control. This is supposed to be due, at least in part, to the curcumin-mediated enhancement of the transcription of the EZH2 3′UTR-*firefly luciferase* gene positioned downstream of the SV40 early enhancer/promoter on the control vector pRL-SV40. Therefore, the co-transfection of the reporter construct p2 and the control vector pRL-SV40 may result in overestimation of the inhibitory effect of curcumin on EZH2 expression in A549 cells.

As previously reported, most of the miRNAs, including miR-101, only lower the expression of their targets by approximately 30%^1^,^2^. We revealed that curcumin up-regulated the expression of miR-let 7c and miR-101 in lung cancer (Wu,G-Q *et al*. submitted for publication). Previous studies reported a negative feedback loop involving EZH2 and miR-let 7c or miR-101^1^,^2^,^3^,^5^,^6^,^7^. In the present study, curcumin caused a 40%-50% decrease in EZH2 3′UTR-luciferase activity depending on the control vector used. The overexpression of miR-let 7c and miR-101 caused an approximately 30% and 40% decrease in EZH2 3′UTR-luciferase activity when tested using p1 and p2, respectively. In addition, the introduction of a transcriptional block by the transcription inhibitor FP revealed that curcumin did not affect the decay of renilla luciferase mRNA transcribed from pRL-SV40. Our interpretation of the result from the dual-luciferase reporter assay using p1 is that in A549 cells transfected with p1, curcumin up-regulates the expression of miR-let 7c and miR-101, which in turn reduces EZH2 3′UTR-luciferase reporter activity by 50–60%. In addition to its inhibitory effect on EZH2 3′UTR-luciferase expression, curcumin promotes the transcription of the EZH2 3′UTR*-luciferase* reporter gene located downstream of the SV40 early enhancer/promoter. However, the effect of the latter far outweighs that of the former, giving rise to the adverse effect of increased EZH2 3′UTR-luciferase activity following curcumin treatment.

In summary, the findings reported here demonstrate that curcumin increases the transcription of *renilla luciferase* when it is positioned downstream of the SV40 early enhancer/promoter but not when it is located downstream of the CMV or HSV-TK promoters. To the best of our knowledge, this is the first report revealing that curcumin enhances the transcription of a gene positioned downstream of the SV40 early enhancer/promoter. It is particularly worth mentioning that the data in this study underscore the need to take caution when interpreting the results of dual-luciferase reporter assays. The enhancer and/or promoter activity, the stability of the mRNA, and protein synthesis may be modified by the agents/molecules being studied. Therefore, to determine the bona-fide effect of an agent/molecule on a 3′UTR, it is strongly recommended that dual-luciferase reporter assays be performed using at least two luciferase reporter vector systems for which the luciferase genes are located upstream of the multiple cloning site (MCS) for the insertion of a 3′UTR and the transcription is driven by different promoters. Alternatively, the absolute copy number of the reporter vector successfully transfected into the target cells can be used as an internal control. The luciferase reporter assay performed with a scrambled or a mutated 3′UTR construct is indispensable.

## Methods

### Plasmid construction

Two hundred and sixty-three base pairs of the EZH2 3′UTR sequence (Accession: NM_004456; bases 2450 to 2712 of the EZH2 transcript variant 1) were amplified by polymerase chain reaction (PCR) using primer set 1 (Table 1) to incorporate Sgf I and Not I restriction sites. The PCR product was inserted into the multiple cloning site (MCS) located downstream of the *renilla luciferase* reporter gene in the dual-luciferase reporter vector pmiR-RB-REPORT^TM^ Vector (RiboBio, Guangzhou, China) to generate p1. To construct p2, 263 bp of the EZH2 3′UTR sequence was amplified by PCR using primer set 2 (Table 1) and cloned into the luciferase reporter vector pMIR-REPORT^TM^ Luciferase (Ambion/Life Technologies, Grand Island, NY) between the Spe I and Hind III sites as previously reported^11^. The artificially synthesized scrambled EZH2 3′UTR was cloned into the MCS of the pmiR-RB-REPORT^TM^ Vector or pMIR-REPORT^TM^ Luciferase to generate the negative control reporter constructs p1-scram and p2-scram, respectively.

### Cell culture and lentiviral transduction

The human lung cancer cell lines A549 and NCI-H2170 were obtained from American Type Culture Collection (ATCC, Manassas, VA). The cells were maintained in RPMI 1640 supplemented with 10% fetal bovine serum (Gibco/Life Technologies, Grand Island, NY) in a humidified atmosphere containing 5% CO_2_ at 37 °C.

A549 cells and NCI-H2170 cells grown in 25-cm^2^ flasks were infected with lentiviral particles expressing miR-let 7c (LV-miR-let 7c), miR-101 (LV-miR-101), or the control virus (LV-Control) (Invitrogen, Shanghai, China) at a multiplicity of infection of 15. Sixteen hours after the transduction, the medium was replaced and the cells were incubated at 37 °C in a humidified atmosphere of 5% CO_2_ for an additional 72 hours before they were used in experiments.

### Luciferase reporter assay

A549 cells were cultured at a density of 2 × 10^4^ cells/well in 96-well culture plates and transfected with 0.2 μg of dual-luciferase reporter construct p1, or co-transfected with 0.2 μg of the luciferase reporter construct p2 and the internal control vector pRL-TK, pRL-SV40, or pRL-CMV (Promega, Madison, WI) at a ratio of 10:1 (reporter construct:control vector) using Lipofectamine^TM^ 2000 (Invitrogen, Carlsbad, CA) according to the manufacturer’s protocol. Six hours post-transfection, the transfection medium was removed and replenished with medium containing 6 μM of curcumin (Sigma-Aldrich, St. Louis, MO) solubilized in 100% dimethylsulfoxide (DMSO, Amresco, Solon, OH) or with medium containing an equivalent volume of DMSO. Forty-eight hours post-transfection, luciferase activity was measured using the Dual-Luciferase^®^ Reporter Assay System (Promega). Renilla luciferase activity was normalized to firefly luciferase activity in cells transfected with the dual-luciferase reporter construct p1, and firefly luciferase activity was normalized to renilla luciferase activity in cells co-transfected with the reporter construct p2 and the control vector.

### qPCR

Forty-eight hours post-transfection, the DNA was isolated from the A549 cells transfected with p1 using the DNeasy Blood & Tissue Kit (Qiagen, Hamburg, Germany) according to the manufacturer’s recommendations. Primer set 3 (Table 1) was designed for the specific amplification of p1 using Vector NTI Advance^®^ 11.5.1. To generate the plasmid standard, the PCR-amplified insert of interest was cloned into the pMD18-T Simple Vector (TaKaRa, Dalian, China) by TA cloning. The 20 μl reactions consisted of 2.0 μl of template DNA (0.5 μg), 2.0 μl of 10 × PCR buffer, 1.2 μl of magnesium (50 mM), 0.5 μl of dNTPs (10 mM), 0.3 μl of SYBR Green 1 Dye (20 × ) (Life Technologies, Carlsbad, CA), 0.2 μl of Taq DNA polymerase, 0.25 μl of the forward and reverse primers of primer set 3, and 13.3 μl of nuclease-free water. The qPCR was carried out on an Applied Biosystems SDS 7500 Fast Instrument (LifeTechnologies, Carlsbad, CA) according to the standard protocol without the 50 °C incubation using version 1.3.1 of the SDS software. The reactions were incubated at 95 °C for 2 min, followed by 40 cycles of 95 °C for 10 s, 60 °C for 30 s, and 70 °C for 45 s. The primers were subjected to a dissociation curve analysis and produced a single peak on a derivative plot of raw fluorescence. The plasmid standard was used to generate a standard curve consisting of five different concentrations. The absolute copy number of p1 was calculated using the standard curve. The qPCR reactions were performed in triplicate for each sample.

For miRNA expression profiling, total RNA was isolated using the RNeasy Mini Kit (Qiagen, Hilden, Germany). TaqMan real-time PCR was carried out as described by the manufacturer (Invitrogen, Shanghai, China) to evaluate the expression of miR-let 7c and miR-101. The relative fold change in miRNA expression was calculated using the 2^−ΔΔCT^ method, where the average of the ^Δ^CT values for the amplicon of interest was normalized to that of U6 and compared with control specimens.

### Transcriptional block

Flavopiridol (FP, MedChem Express, Monmouth Junction, NJ) was employed to induce a transcriptional block. For convenience, FP was prepared as a 10 mM stock solution in DMSO. A549 cells were transfected with pRL-SV40 in 75-cm^2^ flasks using Lipofectamine^TM^ 2000. Twenty-four hours after transfection, the transfected cells were plated in 35-mm dishes at a density of 3 × 10^5^ cells/dish, and FP was added at a final concentration of 200 nM 4 hours after cell plating. Another 4 hours later, curcumin or an equivalent volume of DMSO was added to each dish to obtain a final curcumin concentration of 6 μM. Total RNA was isolated using the RNeasy Mini Kit 24, 48, and 72 hours after the addition of curcumin. qPCR was performed using primer set 4 (Table 1) to determine the relative level of renilla luciferase mRNA whose transcription was driven by the SV40 early enhancer/promoter. The relative fold change in renilla luciferase mRNA expression was calculated using the 2^−ΔΔCT^ method, where the average of the ^Δ^CT values for the renilla luciferase mRNA was normalized to that of beta actin (ACTB, obtained using primer set 5) (Table 1) and compared with control specimens.

### Mann-Whitney-Wilcoxon test

The Mann-Whitney-Wilcoxon test was employed to investigate the differences between the experimental and control arms. *P* < 0.05 was considered statistically significant.

## Additional Information

**How to cite this article**: Wu, G.-Q. *et al*. Evidence for transcriptional interference in a dual-luciferase reporter system. *Sci. Rep.* **5**, 17675; doi: 10.1038/srep17675 (2015).

## Supplementary Material

Supplementary Information

## Figures and Tables

**Table 1:** Primers for vector construction and qPCR analysis.

**Primer**	**Sequence**
Primer set 1	
Forward	5′-GAATTCAAGCGATCGCCATCTGCTACCTCCTCCCCC-3′
Reverse	5′-ATAAGAATGCGGCCGCGACAAGTTCAAGTATTCTTT-3′
Primer set 2	
Forward	5′-CTAGCTAGCATGGGCCAGACTGGGAAGAAAT-3′
Reverse	5′-CGCGGATCCTCAAGGGATTTCCATTTCTCTT-3′
Primer set 3	
Forward	5′-ATGGAAATCCCTTGACATCTGCTA-3′
Reverse	5′-TTGCCCACAGTACTCGAGGTT-3′
Primer set 4	
Forward	5′-ATGACTTCGAAAGTTTATGATCCAG-3′
Reverse	5′-TTTGTTTACATCTGGCCCAC-3′
Primer set 5	
Forward	5′-CTCTGGCCGTACCACTGGC-3′
Reverse	5′-GTGAAGCTGTAGCCGCGC-3′

## References

[b1] Varambally S. . Genomic loss of microRNA-101 leads to overexpression of histone methyltransferase EZH2 in cancer. Science . 322, 1695–1699 (2008). 19008416 10.1126/science.1165395 PMC2684823

[b2] Alajez N. M. . Enhancer of Zeste homolog 2 (EZH2) is overexpressed in recurrent nasopharyngeal carcinoma and is regulated by miR-26a, miR-101, and miR-98. Cell Death Dis . 1, e85 (2010). 21368858 10.1038/cddis.2010.64 PMC3035896

[b3] Zhang J., Guo J. F., Liu D. L., Liu Q. & Wang J. J. MicroRNA-101 exerts tumor-suppressive functions in non-small cell lung cancer through directly targeting enhancer of zeste homolog 2. *J Thorac Oncol*. 6, 671–678 (2011). 21270667 10.1097/JTO.0b013e318208eb35

[b4] Kottakis F. . FGF-2 regulates cell proliferation, migration, and angiogenesis through anNDY1/KDM2B-miR-101-EZH2 pathway. *Mol Cell*. 43, 285–298 (2011). 21777817 10.1016/j.molcel.2011.06.020 PMC3324394

[b5] Au S. L. . Enhancer of zeste homolog 2 epigenetically silences multiple tumor suppressor microRNAs to promote liver cancer metastasis. *Hepatology*. 56, 622–631 (2012). 22370893 10.1002/hep.25679

[b6] Banerjee R. . The tumor suppressor gene rap1GAP is silenced by miR-101-mediated EZH2 overexpression in invasive squamous cell carcinoma. *Oncogene*. 30, 4339–4349 (2011). 21532618 10.1038/onc.2011.141 PMC3154567

[b7] Bao B. . Curcumin analogue CDF inhibits pancreatic tumor growth by switching on suppressor microRNAs and attenuating EZH2 expression. *Cancer Res*. 72, 335–345 (2012). 22108826 10.1158/0008-5472.CAN-11-2182 PMC3792589

[b8] Farr A. & Roman A. A. Pitfall of using a second plasmid to determine transfection efficiency. *Nucleic Acids Res*. 20, 920 (1991). 1311835 10.1093/nar/20.4.920 PMC312047

[b9] Blagosklonny M. V. Flavopiridol, an inhibitor of transcription: implications, problems and solutions. Cell cycle. 3, 1537–1542 (2004). 15539947 10.4161/cc.3.12.1278

[b10] Demidenko Z. N. & Blagosklonny M. V. Flavopiridol Induces p53 via Initial Inhibition of Mdm2 and p21 and, Independently of p53, Sensitizes Apoptosis-Reluctant Cells to Tumor Necrosis Factor. Cancer Res. 64, 3653–3660 (2004). 15150125 10.1158/0008-5472.CAN-04-0204

[b11] Zheng F. . The putative tumour suppressor microRNA-124 modulates hepatocellular carcinoma cell aggressiveness by repressing ROCK2 and EZH2. *Gut*. 61, 278–289 (2012). 21672940 10.1136/gut.2011.239145

