# Supplementary material for: Evidence for transcriptional interference in a dual-luciferase reporter system
Source: Sci Rep. 2015 Dec 1;5:17675. doi: 10.1038/srep17675 (PMC4664949; doi:10.1038/srep17675)
Supplement: Supplementary Information [file srep17675-s1.pdf]

Evidence for transcriptional interference in a dual-luciferase reporter system

Guo-Qing Wu[1*], Xiao Wang[1*], Hong-Ying Zhou[1], Ke-Qun Chai[2], Qian Xue[1], Ai-Hong Zheng[1], Xiu-Ming Zhu[1], Jian-Yong Xiao[3], Xu-Hua Ying[2], Fu-Wei Wang[1], Tao Rui[1], Li-Yun Xu[4], Yong-Kui Zhang[5], Yi-Ji Liao[6], Dan Xie[6], Li-Qin Lu[1], and Dong-Sheng Huang[1]

[1]Department of Oncology & Cancer Biotherapy Center, Zhejiang Provincial People's Hospital, 158 Shangtang Road, Hangzhou, Zhejiang, 310014, China; [2]Zhejiang Academy of Traditional Chinese Medicine, Tongde Hospital of Zhejiang Province, 234 Gucui Road, Hangzhou, Zhejiang, 310012, China; [3]Department of Biochemistry, Guangzhou University of Chinese Medicine, 232 Waihuang Road East, Guangzhou, 510006, China; [4]Cell and Molecular Biology Laboratory, Zhoushan Hospital, Zhejiang 316000, China. [5]Department of Cardio-Thoracic Surgery, Zhoushan Hospital, Zhejiang, 316000, China. [6]State Key Laboratory of Oncology in South China, Cancer Center, Sun Yat-Sen University, 651 Dongfeng Road East, Guangzhou, 510060, Guangdong, China.

[*] Both authors contributed equally to this work.

Address correspondence to: Guo-Qing Wu, Department of Oncology & Cancer Biotherapy Center, Zhejiang Provincial People's Hospital, 158 Shangtang Road, Hangzhou, Zhejiang, 310014, China; Tel: +86-57185893916; Fax: +86-57185131448; gqwuzsu@hotmail.com OR Dong-Sheng Huang, Department of Oncology & Cancer Biotherapy Center, Zhejiang Provincial People's Hospital, 158 Shangtang Road, Hangzhou, Zhejiang, 310014, China; Tel: +86-57185890322; Fax: +86-57185131448; dshuang@zju.edu.cn

This file contains Supplementary Figure S1.

Supplementary Figure S1. FP inhibited cell proliferation and decreased renilla luciferase mRNA levels in pRL-SV40-transfected A549 cells.

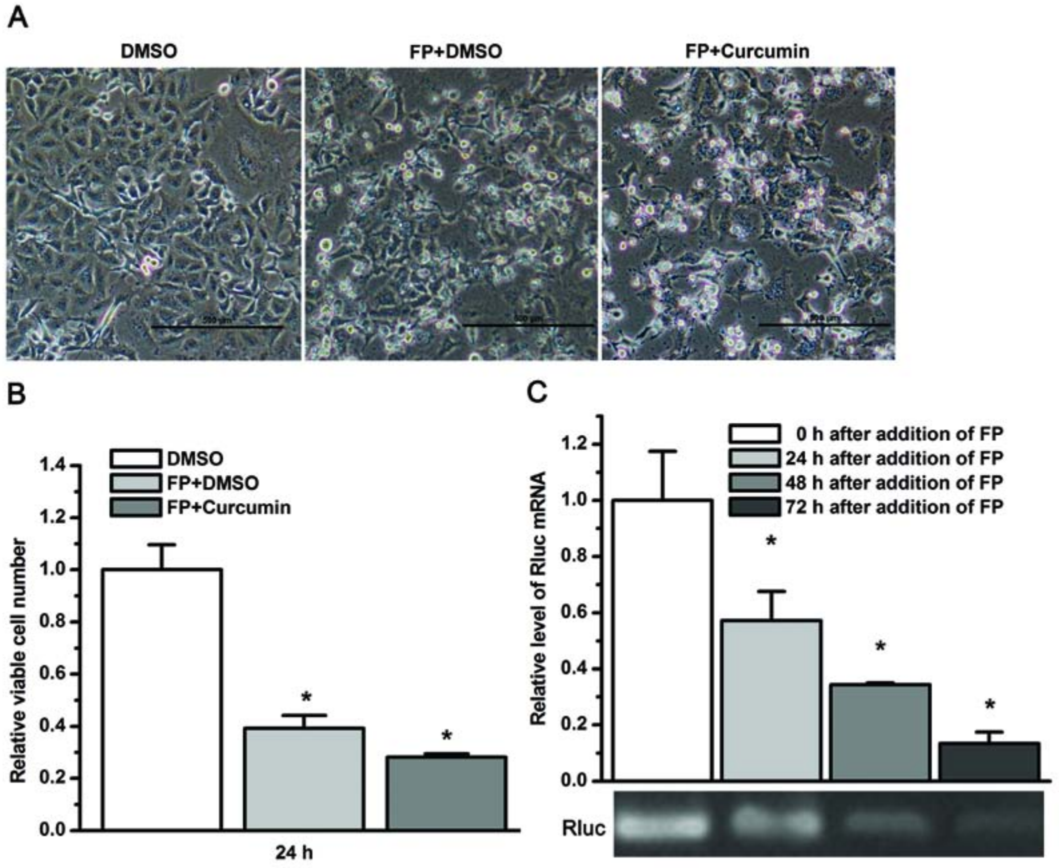

Figure legend

Supplementary Figure S1. FP inhibited cell proliferation and decreased renilla luciferase mRNA levels in pRL-SV40-transfected A549 cells. (A) A549 cells grown for 24 hours in medium containing equivalent volume of DMSO (left panel), 200 nM of FP plus DMSO (middle panel), and 200 nM of FP plus 6 μM of curcumin (right panel). (B) Compared with DMSO, FP plus DMSO or the combination of FP and curcumin significantly inhibited cell proliferation of A549 cells (* $P < 0.05$). The viable cell number of A549 cells treated with FP plus DMSO or FP plus curcumin was normalized to 1 relative to that of control treated with DMSO. (C) FP significantly decreased renilla luciferase mRNA levels time-dependently (* $P < 0.05$). The bar graphs (upper panel) represent renilla luciferase mRNA levels in pRL-SV40-transfected A549 cells treated with FP normalized to that in FP free cells. The bottom panel indicates renilla luciferase mRNA levels determined by reverse transcription-polymerase chain reaction using 1 μg of total RNA as template. Data in all bar graphs were plotted as the mean ± SEM. FP, flavopiridol.
